# Changes in sedentary behaviour in European Union adults between 2002 and 2017

**DOI:** 10.1186/s12889-020-09293-1

**Published:** 2020-08-26

**Authors:** A. López-Valenciano, X. Mayo, G. Liguori, R. J. Copeland, M. Lamb, A. Jimenez

**Affiliations:** 1 Observatory of Healthy & Active Living of Spain Active Foundation, Centre for Sport Studies, King Juan Carlos University, Madrid, Spain; 2 GO fit LAB, Ingesport, Madrid, Spain; 3 University of Rhode Island, Kingston, RI USA; 4 Advanced Wellbeing Research Centre, College of Health, Wellbeing, and Life Sciences, Sheffield Hallam University, Sheffield, UK; 5 The National Centre for Sport and Exercise Medicine, Sheffield, UK; 6 Centre for Behavioural Science and Applied Psychology, Sheffield Hallam University, Sheffield, UK

**Keywords:** Sitting, Sedentarism, National policies, Eurobarometer

## Abstract

**Background:**

Sedentary behaviour (SB) has been identified as an important mortality risk factor. Health organizations have recognised SB as a public health challenge with major health, social, and economic consequences. Researchers have alerted the need to develop specific strategies, to monitor, prevent, and reduce SB. However, there is no systematic analysis of the SB changes in European Union adults. We aimed to examine SB changes between 2002 and 2017 in the European Union (EU) adult population.

**Methods:**

SB prevalence (>4h30mins of sitting time/day) of 96,004 adults as a whole sample and country-by-country was analysed in 2002, 2005, 2013, and 2017 of the Sport and Physical Activity EU Special Eurobarometers’ data. The SB question of a modified version of the International Physical Activity Questionnaire was considered. SB prevalence between countries and within years was analysed with a χ2 test, and SB between genders was analysed with the Z-Score test for two population proportions.

**Results:**

An association between the SB prevalence and the years was found (*p* <  0.001), with increases for the whole sample (2002: 49.3%, 48.5–50.0 95% confidence interval (CI); 2017: 54.5%, 53.9–55.0 95% CI) and men (2002: 51.2%, 50.0–52.4 95% CI; 2017: 55.8%, 55.0–56.7 95% CI) and women (2002: 47.6%, 46.6–48.7 95% CI; 2017: 53.4%, 52.6–54.1 95% CI) separately. The adjusted standardised residuals showed an increase in the observed prevalence versus the expected during 2013 and 2017 for the whole sample and women and during 2017 for men. For all years, differences were observed in the SB prevalence between countries for the whole sample, and men and women separately (*p* <  0.001). Besides, the SB prevalence was always higher in men versus women in the overall EU sample (*p* <  0.001).

**Conclusions:**

SB prevalence increased between 2002 and 2017 for the EU as a whole and for both sexes separately. Additionally, differences in SB prevalence were observed for all years between EU countries in the whole sample and both sexes separately. Lastly, SB was consistently higher in men than women. These findings reveal a limited impact of current policies and interventions to tackle SB at the EU population level.

## Background

Sedentary behaviour (SB) is defined as any waking behaviour characterised by an energy expenditure ≤1.5 metabolic equivalents (METs), while in a sitting, reclining, or lying posture [1]. SB has increased in the industrialised countries in the last decades, with the average adult spending more than half of the day in a SB [2]. This negative lifestyle change presents a major risk factor in the development of many chronic diseases such as obesity, type 2 diabetes, hypertension, cancers, and even premature death [2–5]. In this regard, SB is one of the most important causes of death in developed countries [6]. In European countries, the proportion of deaths attributable to sitting time, a general proxy for SB, is 4.4%, or more than 230,000 deaths/year [7]. Considering this, SB has come to be a major health threat in modern society [8], and awareness of the health and economic burden of SB to policymakers is, therefore, paramount. Men are more frequently sedentary than women [9–11], and independently of the physical activity (PA) performed, SB has negative consequences when sustained for long uninterrupted periods of time [2, 12–14].

The promotion of PA has received substantial and increasing attention globally, with myriad recommendations and plans in circulation [15–17]. By comparison, SB has received limited attention [18]. Previous studies showed that complying with the global recommendations of PA was insufficient to eliminate the increased risk of premature death as a consequence of a high SB (e.g., number of sitting hours) [3, 19], unless the PA occurs at a considerable volume [3, 19], which is difficult to achieve for most of the population. Moreover, Patterson et al. [5] report that the risk of chronic disease associated with SB is not reduced regardless of meeting the recommended PA guidelines. As a consequence, a separate, but equally important focus is required on interventions that help reduce or break-up SB and on public health policy to drive change in SB at a population level [19].

Given the scale of the problem, the World Health Organization (WHO) released a report in 2002, in which it requested countries to develop population-level health promotion strategies to reduce high levels of physical inactivity and sedentary lifestyle. However, there was only a recommendation addressing SB and no specific targets, strategies, or key performance indicators [20].

Since 2002, systematic surveys have been administered to the European Union (EU) member states to monitor SB prevalence with self-report data gathered from the International Questionnaire of Physical Activity (IPAQ) short form. Several studies have analysed SB in these Eurobarometers in a particular year (e.g., 2002 [21], 2005 [9], and 2013 [10, 22]), or as trend data between years [23, 24]. Milton et al., [24] suggested that SB decreased across the EU from 2002 to 2013, while Jelsma et al. (2019) reported that SB was relatively stable over a 15-year period. However, the implication of this time trend analysis was limited by a change in the sitting question included in the Eurobarometer survey between 2005 and 2013 [23, 24]. Each of these studies used the same criteria to determine SB (i.e., >7h30mins), which is typically considered a ‘high’ amount of SB. Therefore, individuals with middle amounts of daily SB (4h31min-7h30mins) were not included. Milton et al. (2015) data showed that merging these two groups increased SB from 51.9% in 2002 to 53% in 2013 [24]. From a public health perspective, it is essential to consider individuals already exceeding 4h30min per day as that is the accepted cut-point resulting in an increased risk of having cardiovascular diseases [25–27] or suffering cardiovascular disease mortality events [28].

With this in mind, is paramount to understand the importance of trends in SB across the EU during the last 15 years, including those who exceed 4h30min/day. Furthermore, data is required to determine the plausible impact of policy development on SB behaviour between those years [29, 30]. This is especially relevant since, through the WHO’s Global Action Plans, it is continued to emphasise the need for strengthening the systems required to implement effective and coordinated actions aiming to reduce SB [16, 17]. A global understanding of SB trends would inform new and update existing policy and position statements in alignment with the recommendations in the global action plan [16, 17].

The primary aim of this study was to identify changes in SB between 2002 and 2017 in EU adults, analysing four separate *Sport and Physical Activity* Eurobarometer’s data. For this, we analysed the SB prevalence (>4h30mins of sitting time/day), considering the between-country differences for all years and the changes within-country between years for the total sample and split by gender. The likely changes were compared against the EU countries’ plans to prevent or reduce SB.

## Methods

### Data source

The European Commission conducts public opinion surveys simultaneously on all EU state members to inquire about the levels of PA, sports participation, and SB among its citizens. These surveys were conducted in 2002, 2005, 2013, and 2017 through the *Sport and Physical Activity* and *Health and Food Special* Eurobarometer’s.

For this study, data were obtained from the adult European population (18–99 years old) of four successive Eurobarometer surveys; December 2002 (Special Eurobarometer 183.6; *n* = 15,363), December 2005 (Special Eurobarometer 246; *n* = 26,413), December 2013 (Special Eurobarometer 412; *n* = 26,988), and December 2017 (Special Eurobarometer 472; *n* = 27,240), with a final sample of *n* = 96,004 (42,546 men and 53,458 women) from the 28 European Union member countries (Austria, Belgium, Bulgaria, Czech Republic, Croatia, Cyprus Republic, Denmark, Estonia, Finland, France, Germany [combined West and East Deutschland], Great Britain, Greece, Hungary, Ireland, Italy, Latvia, Lithuania, Luxembourg, Malta, Netherlands, Poland, Portugal, Romania, Slovakia, Slovenia, Spain, and Sweden). Data from Northern Cyprus and Turkey were not analysed because they do not belong to the EU member countries. Northern Ireland was also not considered due to its unique characteristics.

Eurobarometers use a multi-stage sampling design where primary sampling units are selected from each of the administrative regions in every country. The primary sampling unit’s selection is proportional to the population size of every country, from sampling frames stratified by the degree of urbanization. In this regard, gender, age, region, and the size of the locality were introduced in the iteration procedure. All interviews are conducted face-to-face in people’s homes in their national language [31, 32].

### Measures

The IPAQ is a valid and reliable questionnaire to obtain data on SB [33]. In addition to light, moderate, and vigorous PA, the IPAQ short-form records the total time sitting on an average day as a proxy for SB (i.e., *How much time do you spend sitting on a usual day? This may include time spent at a desk, visiting friends, studying or watching television?*). In the 2002 and 2005 surveys, EU citizens were asked to estimate their usual weekday sitting time using an open-ended response scale. For the 2013 and 2017 surveys, EU citizens were given a choice of 11 categorical response options, ranging from ‘≤ 60 mins’ to ‘>8h30mins’. For this study, to establish a standard measure of SB prevalence in the EU adult population, a cut-off point of 4 h and 30 min was used to define SB (i.e., from ‘>4h30mins’ to ‘> 8h30mins’), as these values show a higher risk of death due to cardiovascular diseases [3, 28]. Furthermore, the close answers in 2013 and 2017 in Eurobarometers did not allow for calculating time spent in SB in relation to other epidemiologic studies, so levels of SB were adapted to these particular categories (i.e., ‘>4h30mins’ and beyond), as Milton et al. (2015) [24]. Individuals answering ‘don’t know’ were included in the analysis.

### Statistical analysis

Descriptive statistics presented as a proportion (%) with the 95% confidence interval (95% CI) were calculated for the SB variable. The SB prevalence within EU countries, for the entire sample and separately for gender and age group (18–24, 25–34, 35–44, 45–54, 55–64, and 65 years and older) were analysed with a χ2 test for 2002, 2005, 2013, and 2017. Additionally, the χ2 test was implemented for comparing behaviour (SB, no-SB, or ‘don’t know’) and years (2002, 2005, 2013, and 2017) along with the analysis of the adjusted standardised residuals. Furthermore, the within-country and within-year differences by gender in SB were analysed using a Z-Score for two population proportions. A priori alpha level was set at 0.05. Z-score analyses were performed with Microsoft Excel version 1709 (Microsoft Corporation; Redmond, Washington, United States of America). Remaining analyses were performed using the Statistical Package for Social Sciences (version 22.0, SPSS Inc., Chicago, IL, USA).

## Results

Significant differences in the prevalence of SB between countries for the entire country sample were observed in 2002 (*n* = 15,363; χ^2^ = 791.963; DF = 28; *p* <  0.001), 2005 (*n* = 26,413; χ^2^ = 1990,145; DF = 54; *p* <  0.001), 2013 (*n* = 26,988; χ^2^ = 1744,015; DF = 52; *p* <  0.001) and 2017 (*n* = 27,240; χ^2^ = 1488,979; DF = 52; *p* <  0.001). Similarly, significant differences between countries were also observed for men in 2002 (*n* = 7082; χ^2^ = 381,420; DF = 28; *p* <  0.001), 2005 (*n* = 11,286; χ^2^ = 1111,757; DF = 54; *p* <  0.001), 2013 (*n* = 12,063; χ^2^ = 828,192; DF = 52; *p* <  0.001) and 2017 (*n* = 12,115; χ^2^ = 777,311; DF = 52; *p* <  0.001); and women in 2002 (*n* = 8281; χ^2^ = 441,942; DF = 28; *p* <  0.001), 2005 (*n* = 15,127; χ^2^ = 1057,698; DF = 54; *p* <  0.001), 2013 (*n* = 14,925; χ^2^ = 1005,487; DF = 52; *p* <  0.001) and 2017 (*n* = 15,125; χ^2^ = 79,778; DF = 52; *p* <  0.001). Descriptive characteristics of the sample can be found in Table 1.

**Table 1:** Descriptive characteristics of the sample

Sample	Overall		2002		2005		2013		2017	
	N	Age	N	Age	N	Age	N	Age	N	Age
**Total**	96,004	50 ± 18	15,363	46 ± 18	26,413	48 ± 18	26,988	50 ± 18	27,240	52 ± 18
**Men**	42,546	50 ± 18	7082	46 ± 17	11,286	46 ± 18	12,063	50 ± 18	12,115	52 ± 18
**Women**	53,458	50 ± 18	8281	46 ± 18	15,127	49 ± 18	14,925	50 ± 17	15,125	52 ± 18

**Fig. 1:**
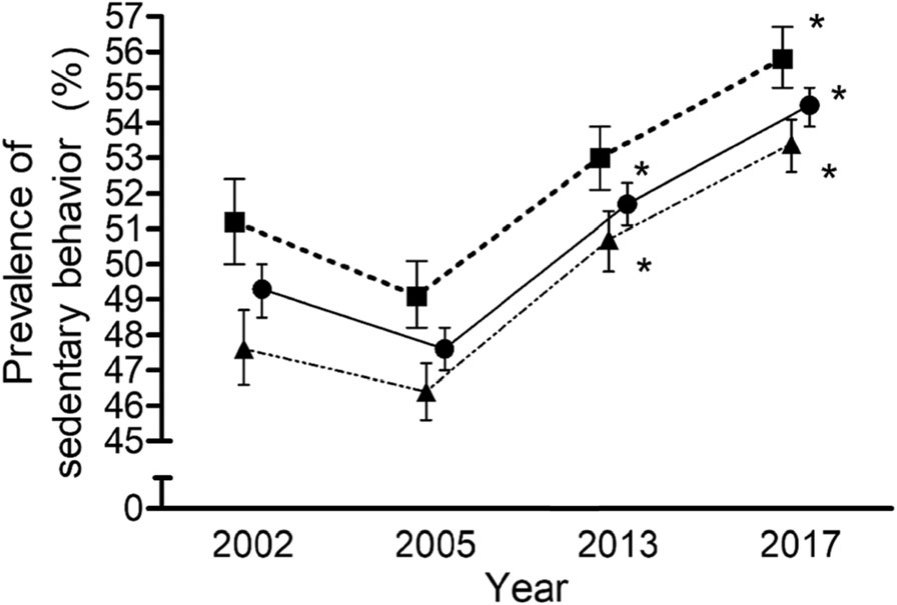
Prevalence (%) of sedentary behaviour (>4h30min/day) in European Union adults (in squares, the men sample; in circles, the whole sample; and in triangles, the women sample) for 2002, 2005, 2013, and 2017. Data are means ± CI. Analysis of the adjusted standardised residuals: *Higher observed prevalence of sedentary behaviour than the expected for the group “>4h30min”

An association between the prevalence of SB and the years were found for the whole sample (*n* = 96,004; χ^2^ = 727,982; DF = 6; *p* <  0.001). These associations were also found for men (*n* = 42,546; χ^2^ = 307,233; DF = 6; *p* <  0.001) and women (*n* = 53,458; χ^2^ = 423,673; DF = 6; *p* <  0.001) separately. As is reflected in Fig. 1, over the 15 year-period in the EU member countries, the adjusted standardized residuals showed an increase in the prevalence observed versus the expected during 2013 and 2017 for the whole sample (adjusted standardized residuals = 2.9 and 13.1) and women (adjusted standardized residuals = 3.1 and 9.8), but only during 2017 for men (adjusted standardized residuals = 8.7). This trend was similar for each of the age groups analysed. Significant differences in the prevalence of SB between age groups for 2002 (χ^2^ = 179,189; DF = 10; *p* <  0.001), 2005 (χ^2^ = 289,434; DF = 10; *p* <  0.001), 2013 (χ^2^ = 184,806; DF = 10; *p* <  0.001) and 2017 (χ^2^ = 161,136; DF = 10; *p* <  0.001) were observed. The SB prevalence for 18–24 and 65 years and older age groups was higher than the expected for all years. Likewise, there were significant differences within age group between years (*p* <  0.001) (Table 2). However, SB prevalence was higher than the expected only for 2013 and 2017 in 35–44 age group, and 2017 in 45–54, 55–64 and 65 years and older age groups.

**Table 2:** Prevalence (%) of sedentary behaviour (>4h30min/day) in European Union (EU) countries adults between 2002 and 2017

Sample	2002			2005			2013			2017			2002–2017	
	Sample	SB (%)	95% CI	Sample	SB (%)	95% CI	Sample	SB (%)	95% CI	Sample	SB (%)	95% CI	χ2	*p*-value
EU^c,d^	*n* = 15,363	49.3	48.4–50.1	*n* = 26,413	47.7	47.1–48.3	*n* = 26,988	51.6	51.0–52.2	*n* = 27,240	54.3	53.7–54.9	683,096	< 0.001
*Age Group*														
18–24	*n* = 1841	57.3	55.0–59.7	*n* = 2642	60.1	58.2–62.0	*n* = 2238	57.5	55.5–59.6	*n* = 1842	58.0	55.5–60.2	53,100	< 0.001
25-34^d^	*n* = 2758	47.0	45.2–48.8	*n* = 4185	47.5	46.1–49.0	*n* = 3793	48.9	47.1–50.4	*n* = 3503	50.5	48.9–52.2	98,418	< 0.001
35-44^c,d^	*n* = 2996	43.7	41.9–45.5	*n* = 4828	43.6	42.2–45.0	*n* = 4467	48.1	46.7–49.5	*n* = 4316	50.4	48.9–51.8	123,955	< 0.001
45-54^d^	*n* = 2549	46.4	44.6–48.5	*n* = 4461	45.1	43.6–46.6	*n* = 4835	49.1	47.8–50.6	*n* = 4613	51.7	50.2–53.2	96,233	< 0.001
55-64^d^	*n* = 2267	47.6	45.7–49.8	*n* = 4486	44.5	43.1–45.9	*n* = 5013	49.6	48.2–50.9	*n* = 5019	53.2	51.9–54.7	136,982	< 0.001
65 years and older^d^	*n* = 2952	55.8	54.1–57.5	*n* = 5811	50.2	48.9–51.5	*n* = 6642	56.8	55.6–57.9	*n* = 7947	59.4	58.3–60.4	266,122	< 0.001
*Countries*														
Austria^c,d^	*n* = 979	40.0	37.0–43.3	*n* = 981	55.4	52.4–58.4	*n* = 1006	59.2	56.0–62.1	*n =* 1011	63.1	59.9–66.1	276,263	< 0.001
Belgium^d^	*n* = 1065	47.8	44.7–50.8	*n* = 964	54.6	51.1–57.6	*n* = 1047	55.7	52.5–58.7	*n =* 985	58.7	55.7–61.6	193,779	< 0.001
Bulgaria^c,d^				*n* = 953	43.2	39.9–46.3	*n* = 1007	57.9	54.8–60.9	*n =* 1015	55.7	52.6–58.9	61,226	< 0.001
Croatia^c^				*n* = 966	48.2	44.9–51.1	*n* = 992	55.1	52.1–58.3	*n =* 1018	51.2	47.9–54.3	9977	0.041
Cyprus				*n* = 473	52.9	48.4–57.3	*n* = 483	52.0	47.6–56.3	*n* = 487	47.4	42.7–51.7	68,164	< 0.001
Czech Republic				*n* = 995	58.0	55.1–61.2	*n* = 998	59.5	56.5–62.4	*n =* 1011	62.1	59.3–64.9	71,718	< 0.001
Denmark^c^	*n* = 988	67.1	64.1–69.9	*n* = 1011	63.6	60.5–66.7	*n* = 992	71.7	68.9–74.4	*n =* 996	67.3	64.3–70.4	52,219	< 0.001
Estonia^d^				*n* = 955	52.0	49.0–55.3	*n* = 993	55.7	52.4–58.8	*n =* 986	60.8	57.9–63.8	43,981	< 0.001
Finland^a,c^	*n* = 977	61.4	58.5–64.6	*n* = 982	52.2	49.6–55.4	*n* = 954	61.5	58.2–64.5	*n =* 1008	53.0	50.0–55.9	48,861	< 0.001
France^c,d^	*n* = 1011	43.3	40.5–46.3	*n* = 986	40.7-	37.7–43.7	*n* = 1002	49.1	45.8–52.0	*n =* 991	51.0	47.6–54.2	66,148	< 0.001
Germany^d^	*n* = 1991	50.0	48.0–52.3	*n* = 1512	50.6	48.1–53.2	*n* = 1566	51.3	48.9–53.8	*n =* 1577	53.7	51.0–56.2	148,307	< 0.001
Great Britain^c,d^	*n* = 984	43.5	40.2–46.9	*n* = 989	46.9	43.7–50.1	*n* = 975	56.4	53.3–59.5	*n =* 1015	53.3	50.3–56.2	166,157	< 0.001
Greece^d^	*n* = 969	48.4	45.3–51.9	*n* = 979	69.8	66.9–72.8	*n* = 975	55.9	52.8–59.3	*n =* 979	63.7	60.6–66.4	121,256	< 0.001
Hungary^d^				*n* = 990	37.2	34.1–40.3	*n* = 997	38.8	35.9–41.8	*n =* 1033	46.7	43.6–49.5	31,043	< 0.001
Italy^a,d^	*n* = 995	53.7	50.8–56.7	*n* = 969	43.7	40.5–46.5	*n* = 1009	43.3	40.3–46.3	*n =* 1026	53.8	50.9–56.8	47,827	< 0.001
Ireland^d^	*n* = 955	40.3	37.4–43.6	*n* = 968	37.9	34.8–40.9	*n* = 979	42.3	39.2–45.7	*n =* 985	47.7	44.7–50.8	143,304	< 0.001
Latvia^c^				*n* = 933	42.7	39.4–45.8	*n* = 979	50.8	47.8–53.8	*n =* 971	49.2	46.0–52.5	30,446	< 0.001
Lithuania^c,d^				*n* = 958	35.8	32.8–38.8	*n* = 980	51.9	48.8–55.0	*n =* 998	50.0	47.1–53.3	216,693	< 0.001
Malta^d^				*n* = 483	29.0	25.3–33.3	*n* = 496	35.9	31.7–39.9	*n =* 500	47.2	42.6–51.6	91,250	< 0.001
Luxembourg	*n* = 580	49.8	45.5–54.0	*n* = 472	48.1	43.6–52.5	*n* = 490	52.9	48.6–57.6	*n =* 485	52.4	47.8–56.9	33,608	< 0.001
Poland^c^				*n* = 950	52.2	48.9–55.4	*n* = 986	43.4	40.5–46.7	*n =* 981	45.5	42.3–48.5	41,338	< 0.001
Portugal^d^	*n* = 949	31.0	28.0–33.9	*n* = 968	24.9	22.1–27.4	*n* = 1037	34.1	31.3–36.9	*n =* 1068	44.3	41.4–47.2	156,497	< 0.001
Romania^d^				*n* = 960	28.7	25.7–31.7	*n* = 987	36.0	32.9–39.1	*n =* 974	40.6	37.3–43.7	59,108	< 0.001
Slovakia^d^				*n* = 1029	47.9	45.0–51.2	*n* = 979	55.3	51.9–58.2	*n =* 1086	56.7	53.9–59.7	63,072	< 0.001
Slovenia^b,d^				*n* = 985	46.9	43.6–50.1	*n* = 1096	35.0	32.2–37.8	*n =* 1016	45.7	42.8–48.6	71,376	< 0.001
Spain	*n* = 938	44.1	40.9–47.3	*n* = 987	41.0	38.0–44.0	*n* = 990	43.3	40.3–46.8	*n =* 1002	45.8	42.7–48.9	114,461	< 0.001
Sweden^c,d^	*n* = 987	57.4	54.2–60.4	*n* = 1021	54.1	50.7–57.3	*n* = 991	65.7	62.7–68.7	*n =* 1034	67.8	64.9–70.9	81,934	< 0.001
The Netherlands^c,d^	*n* = 995	59.3	56.4–62.4	*n* = 994	67.5	64.7–70.6	*n* = 1002	73.7	70.9–76.2	*n =* 1002	79.9	77.4–82.4	171,853	< 0.001

*CI* Confidence intervals. Analysis of the adjusted standardised residuals: Higher observed cases than the expected on the >4h30min box for 2002 (a), 2005 (b), 2013 (c), and 2017 (d)

In 2004, the number of EU countries increased from 15 to 28. Therefore, an additional analysis was performed only considering the first 15 countries. For this group of countries, an association between SB prevalence and the years were found for the whole sample (*n* = 60,325; χ2 = 661,052; DF = 6; *p* <  0.001). The analysis of the residuals showed an increase in the prevalence of SB observed versus the expected during 2013 and 2017 (adjusted standardised residuals = 4.5 and 12.3). These differences were also found for men (*n* = 28,060; χ2 = 333,673; DF = 6; *p* <  0.001) and women (*n* = 32,265; χ2 = 329,483; DF = 6; *p* <  0.001) separately. An increase was also reported in the prevalence of SB observed versus the expected during 2013 and 2017 for men (adjusted standardised residuals = 3.2 and 8.2) and women (adjusted standardised residuals = 3.1 and 9.0).

All the countries showed changes in SB prevalence between years (Table 2), with most of them showing an observed higher prevalence in 2017 than the expected (i.e., Austria, Belgium, Bulgaria, Estonia, France, Germany, Great Britain, Greece, Hungary, Italy, Ireland, Lithuania, Malta, Portugal, Romania, Slovakia, Slovenia, Sweden, and The Netherlands). Only Finland showed fewer observed cases than the expected for 2017.

While considering the subsamples of men and women separately for every country, and as can be observed in Table 3, similar patterns are generally reported. Differences between years were observed for most of the countries except for men in Croatia. The SB prevalence observed in 2017 was higher than the expected for men in Austria, Germany, Great Britain, Hungary, Ireland, Lithuania, Portugal, Romania, Slovakia, Sweden, and The Netherland. For women, the increase in the cases reported versus the expected was observed for Belgium, Bulgaria, France, Greece, Hungary, Italy, Ireland, Malta, Portugal, Romania, Slovenia, Sweden, and The Netherland.

**Table 3:** Prevalence (%) of sedentary behaviour (>4h30min/day) in men and women of European Union (EU) countries and gender differences between 2002 and 2017

	Gender	2002					2005					2013					2017					2002–2017	
		Sample	SB (%)	95% CI	*Z*-score	*p*-value	Sample	SB (%)	95% CI	*Z*-score	*p*-value	Sample	SB (%)	95% CI	*Z*-score	*p*-value	Sample	SB (%)	95% CI	Z-score	p-value	χ2	*p*-value
EU	Men^d^	*n* = 7082	51.2	50.0–52.4	4.59	< 0.001	*n* = 11,286	49.4	48.5–50.3	4.67	< 0.001	*n* = 12,063	52.7	51.8–53.7	3.27	< 0.001	*n* = 12,115	55.8	54.9–56.8	4.45	< 0.001	311,002	< 0.001
	Women^c,d^	*n* = 8281	47.6	46.6–48.7			*n* = 15,127	46.5	45.8–47.3			*n* = 14,925	50.7	49.8–51.5			*n* = 15,125	53.1	52.2–53.8			374,081	< 0.001
*Country*																							
Austria	Men^c,d^	*n* = 387	41.1	36.2–46.0	0.54	0.59	*n* = 477	50.7	45.9–55.3	2.83	< 0.001	*n* = 476	58.6	53.8–63.0	0.33	0.74	*n* = 477	65.4	61.2–69.6	1.43	0.15	137,597	< 0.001
	Women^b,c,d^	*n* = 592	39.4	35.6–43.4			*n* = 504	59.7	55.6–63.9			*n* = 530	59.6	55.5–63.6			*n* = 534	61.1	56.9–65.5			149,116	< 0.001
Belgium	Men^b^	*n* = 510	49.6	45.1–53.5	1.14	0.26	*n* = 469	60.6	56.3–65.0	3.64	< 0.001	*n* = 507	58.6	54.2–63.1	1.83	0.07	*n* = 466	58.4	53.6–62.9	0.19	0.85	100,024	< 0.001
	Women^d^	*n* = 555	46.1	42.0–50.3			*n* = 495	48.9	44.7–53.1			*n* = 540	53.0	48.5–57.6			*n* = 519	59.0	55.1–63.4			102,617	< 0.001
Bulgaria	Men^c^						*n* = 452	37.8	33.2–42.7	3.20	< 0.001	*n* = 468	54.9	50.2–59.2	1.79	0.07	*n* = 459	50.3	45.8–54.9	3.11	< 0.001	39,992	< 0.001
	Women^c,d^						*n* = 501	48.1	44.1–52.5			*n* = 539	60.5	56.4–64.7			*n* = 556	60.1	55.9–63.8			25,428	< 0.001
Croatia	Men						*n* = 395	50.4	45.3–54.9	1.11	0.27	*n* = 439	55.6	50.8–60.4	0.25	0.80	*n* = 442	55.4	51.1–60.0	2.38	0.02	4711	0.318
	Women^c^						*n* = 571	46.8	42.7–51.0			*n* = 553	54.8	50.6–59.0			*n* = 576	47.9	44.1–52.1			10,229	0.037
Cyprus	Men^c^						*n* = 198	57.1	50.0–63.1	1.57	0.11	*n* = 225	52.4	45.8–59.1	0.18	0.86	*n* = 216	52.8	45.8–59.3	2.11	0.04	35,839	< 0.001
	Women^c^						*n* = 275	49.8	44.0–55.6			*n* = 258	51.6	45.3–57.4			*n* = 271	43.2	37.3–49.1			35,577	< 0.001
Czech Republic	Men						*n* = 459	58.8	54.0–63.4	0.49	0.62	*n* = 418	61.5	56.9–66.0	1.07	0.28	*n* = 430	61.6	57.2–66.3	0.28	0.78	24,993	< 0.001
	Women						*n* = 536	57.3	53.0–61.2			*n* = 580	58.1	54.7–62.1			*n* = 581	62.5	58.5–66.4			49,404	< 0.001
Denmark	Men	*n =* 489	66.1	62.0–70.3	0.70	0.48	*n* = 523	66.9	62.9–70.9	2.27	0.02	*n* = 483	72.1	67.9–75.8	0.26	0.80	*n* = 510	69.0	65.3–73.1	1.21	0.23	31,976	< 0.001
	Women^c^	*n =* 499	68.1	63.9–72.1			*n* = 488	60.0	55.5–64.5			*n* = 509	71.3	67.6–75.0			*n* = 486	65.4	60.9–69.7			32,921	< 0.001
Estonia	Men^d^						*n* = 311	49.5	44.1–55.3	1.08	0.28	*n* = 399	54.6	49.6–59.4	0.55	0.58	*n* = 344	59.0	54.1–64.2	0.82	0.41	36,826	< 0.001
	Women^d^						*n* = 644	53.3	49.4–57.1			*n* = 594	56.4	52.5–60.4			*n* = 642	61.7	57.9–65.4			16,464	0.002
Finland	Men^a^	*n =* 414	63.3	58.7–68.4	1.03	0.30	*n* = 399	56.1	51.1–61.4	2.02	0.04	*n* = 429	61.5	57.1–66.2	0.00	1.00	*n* = 493	54.2	49.5–58.6	0.74	0.46	17,986	0.006
	Women^c^	*n =* 563	60.0	56.0–64.1			*n* = 583	49.6	45.5–53.7			*n* = 525	61.5	57.5–65.7			*n* = 515	51.8	47.4–56.1			33,536	< 0.001
France	Men^d^	*n =* 480	46.3	42.1–50.6	1.79	0.07	*n* = 444	42.1	37.8–46.8	0.84	0.40	*n* = 461	49.9	45.3–54.4	0.46	0.64	*n* = 433	54.0	49.7–58.7	1.71	0.09	35,693	< 0.001
	Women^c,d^	*n =* 531	40.7	36.2–45.2			*n* = 542	39.5	35.1–43.7			*n* = 541	48.4	44.0–52.5			*n* = 558	48.6	44.4–52.7			37,430	< 0.001
Germany	Men^d^	*n =* 939	49.5	46.1–52.5	0.49	0.62	*n* = 678	54.4	50.9–58.1	2.69	0.01	*n* = 770	52.5	49.1–56.1	0.88	0.38	*n* = 776	57.2	53.6–60.8	2.80	0.01	81,819	< 0.001
	Women	*n =* 1052	50.8	47.5–53.6			*n* = 834	47.5	44.2–51.0			*n* = 796	50.3	46.7–53.5			*n* = 801	50.2	46.7–53.7			74,897	< 0.001
Great Britain	Men^c,d^	*n =* 333	45.7	40.5–51.1	0.97	0.33	*n* = 469	48.2	43.5–53.1	0.76	0.45	*n* = 458	60.7	56.3–65.3	2.54	0.01	*n* = 505	57.2	52.9–61.4	2.50	0.01	83,335	< 0.001
	Women^c^	*n =* 651	42.4	38.6–46.2			*n* = 520	45.8	41.7–49.8			*n* = 517	52.6	48.0–56.9			*n* = 510	49.4	44.7–53.5			78,646	< 0.001
Greece	Men^b^	*n =* 481	49.9	44.9–53.6	0.67	0.50	*n* = 417	67.2	62.6–71.9	1.54	0.12	*n* = 469	59.5	54.8–64.0	2.17	0.03	*n* = 442	62.9	58.6–67.4	0.50	0.62	39,455	< 0.001
	Women^b,d^	*n =* 488	47.3	42.8–51.4			*n* = 562	71.7	67.8–75.4			*n* = 506	52.6	48.2–57.1			*n* = 537	64.4	60.3–68.5			89,735	< 0.001
Hungary	Men^d^						*n* = 388	35.1	30.2–39.9	1.11	0.27	*n* = 412	35.0	30.6–39.6	2.10	0.04	*n* = 425	44.2	39.8–49.2	1.31	0.19	14,959	0.005
	Women^d^						*n* = 602	38.5	34.9–42.4			*n* = 585	41.5	37.6–45.6			*n* = 608	48.4	44.4–52.8			17,964	0.001
Italy	Men^a^	*n =* 481	55.3	50.5–59.7	1.00	0.32	*n* = 371	44.5	39.4–49.9	0.41	0.68	*n* = 444	47.1	42.8–51.6	2.14	0.03	*n* = 509	51.5	47.2–56.0	1.48	0.14	14,705	0.023
	Women^a,d^	*n =* 514	52.1	47.7–56.4			*n* = 598	43.1	39.1–47.0			*n* = 565	40.4	36.1–44.4			*n* = 517	56.1	52.0–60.3			39,341	< 0.001
Ireland	Men^d^	*n =* 461	40.1	35.8–44.5	0.06	0.95	*n* = 427	42.2	37.5–47.1	2.42	0.02	*n* = 429	49.0	44.1–53.4	3.73	< 0.001	*n* = 471	52.4	48.2–56.9	2.84	< 0.001	100,620	< 0.001
	Women^d^	*n =* 494	40.5	36.0–44.9			*n* = 541	34.6	30.3–38.4			*n* = 550	37.1	33.1–41.1			*n* = 514	43.4	39.3–47.5			56,911	< 0.001
Latvia	Men						*n* = 324	37.4	32.4–42.9	2.39	0.02	*n* = 446	48.4	43.7–53.1	1.34	0.18	*n* = 358	48.0	42.7–53.9	0.57	0.57	20,231	< 0.001
	Women						*n* = 609	45.5	41.9–49.3			*n* = 533	52.7	48.6–56.8			*n* = 613	49.9	46.2–53.7			13,730	0.008
Lithuania	Men^c,d^						*n* = 353	30.3	25.2–35.1	2.71	0.01	*n* = 442	49.3	44.8–54.1	1.49	0.14	*n* = 367	49.1	44.1–54.0	0.46	0.65	113,428	< 0.001
	Women^c^						*n* = 605	39.0	35.2–43.1			*n* = 538	54.1	49.8–58.2			*n* = 631	50.6	46.9–54.5			110,817	< 0.001
Luxembourg	Men	*n =* 272	55.9	50.0–61.8	2.74	0.01	*n* = 194	57.7	51.0–64.4	3.50	< 0.001	*n* = 197	63.6	56.9–70.1	3.85	< 0.001	*n* = 196	57.1	50.5–63.8	1.73	0.08	18,303	0.006
	Women	*n =* 308	44.5	39.0–50.0			*n* = 278	41.4	35.6–47.1			*n* = 293	45.7	40.3–51.5			*n* = 289	49.1	43.3–55.0			18,322	0.005
Malta	Men						*n* = 157	43.3	36.3–51.0	4.82	< 0.001	*n* = 199	40.7	34.2–47.7	1.83	0.07	*n* = 211	47.4	40.8–53.6	0.07	0.94	36,146	< 0.001
	Women^d^						*n* = 326	22.1	18.1–26.4			*n* = 297	32.7	27.6–38.0			*n* = 289	47.1	41.5–52.9			68,420	< 0.001
Poland	Men^a^						*n* = 413	51.6	47.0–56.7	0.35	0.73	*n* = 382	42.2	36.9–47.4	0.64	0.52	*n* = 396	48.7	43.7–53.8	1.69	0.09	22,746	< 0.001
	Women^a^						*n* = 537	52.7	48.4–57.0			*n* = 604	44.2	40.2–48.3			*n* = 585	43.3	39.1–47.2			22,650	< 0.001
Portugal	Men^d^	*n =* 432	33.3	28.9–38.2	1.43	0.15	*n* = 387	26.9	22.2–31.3	1.16	0.25	*n* = 461	37.7	33.0–42.1	2.19	0.03	*n* = 435	47.1	42.5–51.7	1.55	0.12	63,050	< 0.001
	Women^d^	*n =* 517	29.0	25.3–33.1			*n* = 581	23.6	20.3–27.0			*n* = 576	31.3	27.4–35.1			*n* = 633	42.3	38.4–46.1			97,001	< 0.001
Romania	Men^d^						*n* = 432	30.1	26.2–34.7	0.90	0.37	*n* = 498	35.3	31.3–39.8	0.41	0.68	*n* = 460	41.7	36.7–45.9	0.71	0.48	31,476	< 0.001
	Women^d^						*n* = 528	27.5	23.7–31.1			*n* = 489	36.6	32.3–40.9			*n* = 514	39.5	34.8–43.6			30,620	< 0.001
Slovakia	Men^d^						*n* = 392	44.9	39.8–49.7	1.52	0.13	*n* = 414	55.8	51.0–60.4	0.29	0.77	*n* = 472	58.5	54.0–63.1	1.02	0.31	38,269	< 0.001
	Women						*n* = 637	49.8	46.0–53.7			*n* = 565	54.9	50.4–58.9			*n* = 614	55.4	51.3–59.4			31,092	< 0.001
Slovenia	Men^b^						*n* = 434	47.7	43.1–52.1	0.44	0.66	*n* = 455	35.4	31.2–40.0	0.26	0.80	*n* = 463	43.0	38.7–47.3	1.58	0.11	25,593	< 0.001
	Women^b,d^						*n* = 551	46.3	41.9–50.3			*n* = 641	34.6	31.0–38.2			*n* = 553	47.9	43.6–52.1			48,760	< 0.001
Spain	Men	*n =* 452	43.8	39.2–48.7	0.19	0.85	*n* = 422	42.7	37.9–46.9	0.89	0.37	*n* = 464	46.3	41.8–50.4	1.79	0.07	*n* = 447	48.8	44.1–53.5	1.69	0.09	67,644	< 0.001
	Women	*n =* 486	44.4	40.5–49.0			*n* = 565	39.8	36.1–43.7			*n* = 526	40.7	36.7–45.2			*n* = 555	43.4	39.5–47.7			50,332	< 0.001
Sweden	Men^d^	*n =* 464	60.8	56.5–64.7	2.05	0.04	*n* = 563	56.0	51.9–60.0	1.34	0.18	*n* = 503	65.4	61.0–69.4	0.19	0.85	*n* = 543	66.9	63.0–70.9	0.68	0.49	33,950	< 0.001
	Women^c,d^	*n =* 523	54.3	49.9–58.7			*n* = 458	51.8	47.2–56.3			*n* = 488	66.0	62.1–70.1			*n* = 491	68.8	65.2–72.7			53,305	< 0.001
The Netherlands	Men^d^	*n =* 487	66.5	62.4–70.6	4.55	< 0.001	*n* = 493	74.0	70.0–78.1	4.36	< 0.001	*n* = 458	74.2	70.5–77.9	0.38	0.70	*n* = 533	82.0	79.2–85.2	1.73	0.08	52,244	< 0.001
	Women^c,d^	*n =* 508	52.4	48.0–56.7			*n* = 501	61.1	57.1–65.3			*n* = 544	73.2	69.3–76.8			*n* = 469	77.6	73.8–81.2			129,678	< 0.001

*CI* Confidence intervals. Analysis of the adjusted standardised residuals: Higher observed cases than the expected on the >4h30min box for 2002 (a), 2005 (b), 2013 (c), and 2017 (d)

When analysing gender differences (Table 3), SB prevalence in the overall EU sample was significantly higher in men compared to women for the whole sample. Almost all countries displayed greater SB prevalence in men in comparison with women over the years, with the following exceptions showing higher levels of SB prevalence in women in 2002 (Germany, Denmark, Ireland, and Spain), 2005 (Austria, Bulgaria, Estonia, Greece, Hungary, Latvia, Lithuania, Poland, and Slovakia), 2013 (Austria, Bulgaria, Estonia, Hungary, Latvia, Lithuania, Poland, Romania, and Sweden), and 2017 (Belgium, Bulgaria, Czech Republic, Estonia, Greece, Hungary, Italy, Latvia, Lithuania, Slovenia, and Sweden). Only five countries have shown a greater SB prevalence in women versus men for all the years (Bulgaria, Estonia, Hungary, Latvia, and Lithuania).

## Discussion

The main findings were that (a) there was a recurrent difference between countries for all years, indicating that there is a dissimilar capability to prevent or reduce the prevalence of SB across the EU; (b), there was an increase in SB prevalence in the European adults from 2005 to 2017 considering the whole sample and men and women separately; and (c) there was a generally higher prevalence of SB in men than women, with a similar descriptive trend from 2005 to 2017.

Previous studies have reported that SB was rather stable over the 15-year period [23], or even declined based on 2002, 2005, and 2013 Eurobarometer data [24]. Nevertheless, there is an important difference to consider when comparing our data with the findings of the previous studies. Jelsma et al. (2019) [23] analysed only the percentages of population with more than 7h30mins per day of SB, while Milton et al. (2015) established conclusions with the data of the high sitting group (> 7h30min) when the middle sitting group (> 4 h31 to 7 h30 min) was not included in the analysis [24]. When considering the 4h30min group, there exists a trend of increasing SB prevalence over the years, similarly to ours. This discrepancy is very relevant to consider when analysing the information provided by each of these studies since it could lead to different outcomes. In our opinion, considering individuals with >4h30mins is pertinent because different studies have already shown an increased risk of suffering cardiovascular diseases and premature death in people who accumulate more than 4 h daily of SB [3, 19]. While it is clear that increased hours of SB results in worsening health outcomes, reducing individual-level SB time, for all individuals, yields the greatest overall public health benefit. For example, as reducing sitting time by ~ 2 h/day results in a 2.3% decrease in mortality [7].

This increase in the prevalence of SB could be explained by the social and environmental changes. For example, longer work commute durations, a greater number of labour-saving devices both at home and work [34] and urban environment inequalities that force people to travel longer distances and live in areas that lack support for active lifestyles [35] could all be contributing to the increased SB time. Furthermore, work and leisure-time are related to technology and consequently, people of all ages are spending more time interacting with technology in the form of Internet, videogames, interactive television, mobile phones, etc. [36].

Policy development on SB prevention has received increased attention in the last decade [18]. Some general recommendations from national and international organisations began to emerge at the end of the 2000s for reducing SB, such as the example the *EU Physical Activity Guidelines* [37] or the *Physical Activity and Health Report* from the U.S. [38], and most notably the World Health Organization supporting evidence to action through the *Physical Activity and Health in Europe* [39]. Policy-level interventions to reduce SB are, however, less developed than those attempting to reduce population levels of physical inactivity [40]. A previous analysis review found that only 22% of PA guidelines mentioned SB as part of a policy [41]. Besides, another study showed that very few countries had documents related to SB independently of PA policies [42]. This is despite evidence that suggests SB has more influence on decreasing health outcomes compared to physical inactivity [43]. In this sense, some countries may have more recently developed SB policies. In contrast, others still do not have any defined guidelines, aim, or even specific surveillance and monitoring systems that could help reduce SB.

In line with early calls to introduce public health guidelines on SB as soon as possible [44], some countries have made attempts to develop a policy regarding SB such as Belgium [45], France [46], Germany [47], Great Britain [48, 49], Spain [50], Sweden [51], and the Netherlands [52]. Still, a greater focus across all EU countries is required. This needs to extend to include appropriate surveillance and monitoring systems that assess attempts to reduce SB as well as guidelines themselves. This has been identified recently [53], underlining the importance of the evidence base when developing prescriptive public health guidance on SB as once established, and they are difficult to modify without generating confusion – as seen with the PA guidelines [54].

Regarding gender differences, results are consistent with previous studies where the prevalence of SB was always higher in men than women [9, 23, 24, 55]. Previous studies have shown that regarding gender, SB might be context-dependent [22]. For example, highly educated individuals spend more time sitting, which is still the case for more men than women in some EU countries, particularly those in Eastern Europe [9]. On the other hand, older women have been shown to be less sedentary than older men, probably because they still spend more time on household activities [56]. An alternative explanation could be related to the pattern of SB, in which women are more likely to accumulate their sedentary time in shorter bouts and, therefore, more likely to break up prolonged periods of sitting than men [56]. The consistent finding of higher SB prevalence in men should be an important point of consideration when discussing policy for SB reduction efforts.

Some limitations of this study should be recognised. Firstly, methodological differences exist between 2002 and 2005 and 2013–2017 data collection, which was solved using the same cut-points for each of the 4 years data were collected. Secondly, SB was assessed using a single recall item focused on one typical day, yet SB oscillates greatly from 1 day to another. Eurobarometer data may, therefore, underestimate sitting time when compared to an objective tool such as accelerometry, which is the gold standard for SB [10, 57–59]. Lastly, our study did not contemplate specific patterns of SB regarding breaking time of SB while standing, stretching, or including light PA, which might have different effects on the individuals.

Despite general efforts internationally to reduce SB, current data make clear the need for strengthening existing policies and developing new ones to address SB prevalence. Although numerous studies acknowledge the hazards of excessive SB, there are very few specific SB recommendations at a population level. Moreover, guidelines should target SB independently of PA, with specific goals and key performance indicators identified to reduce SB [42]. SB is arguably an easier behaviour to perform than PA, because no equipment is required, and it can be as simple as a person standing. It has been acknowledged that reducing SB is the first step on the physical activity behavioral continuum [60], meaning that changes to SB could also facilitate increases in PA in the future. Policies would need to make clear to the public how to reduce SB in tangible ways. Policies also need to articulate the difference between SB and PA clearly. Secondly, countries with SB defined policies should assess and strengthen said policies, monitoring surveillance data, and evaluating previous and ongoing interventions [16]. Countries without policies should develop plans on SB, following current recommendations, and learning from others that have shown even moderate success [16, 42]. Finally, none of the EU countries considered gender in their written policy, yet it is clear that gender differences exist in the volume and pattern of SB [56].

## Conclusions

There were differences in SB prevalence between EU countries for all the years when considering the whole sample and for men and women separately, indicating an unequal capacity for tackling sedentary behaviour in the continent. Additionally, and considering the last 15 years of SB monitoring, an increase in SB for EU adults was observed both as a whole and while considering genders separately, indicating a limited impact of existing SB policy. Lastly, a generally higher SB prevalence in men than women is usually reported, remaining consistent over time. Futures analyses should be implemented across EU with objective measures of SB.

## Data Availability

The raw data is owned by the European Commission and available online (Special Eurobarometer 183–6, December 2002: https://dbk.gesis.org/ dbksearch/sdesc2.asp?no=3886&search=58.2&search2=&field=all& field2=all&DB=e&tab=0&notabs=&nf=1&af=&ll=10. Special Eurobarometer 246, December 2005: https://dbk.gesis.org/dbksearch/sdesc2.asp?no=4415& search=64.3&search2=&field=all&field2=&DB=e&tab=0&notabs=& nf=1&af=&ll=10. Special Eurobarometer 412, March 2014: https://dbk.gesis.org/dbksearch/sdesc2.asp?no=5877&search=Physical%20fitness%20and%20exercise&search2=&field=all&field2=&DB=e&tab=0&notabs=&nf=1&af=&ll=10. Special Eurobarometer 472, March 2018: https://dbk.gesis.org/dbksearch/sdesc2.asp?no=6939&search=Physical%20fitness%20and%20exercise&search2=&field=all&field2=&DB=e&tab=0&notabs=&nf=1&af=&ll=10).

## Abbreviations

CI: Confidence interval
IPAQ: International Physical Activity Questionnaire
PA: Physical activity
SB: Sedentary behaviour
WHO: World Health Organization
